# Supplementary material for: Intensified continental chemical weathering and carbon-cycle perturbations linked to volcanism during the Triassic–Jurassic transition
Source: Nat Commun. 2022 Jan 13;13:299. doi: 10.1038/s41467-022-27965-x (PMC8758789; doi:10.1038/s41467-022-27965-x)
Supplement: Supplementary file 1 — Supplementary Information [file 41467_2022_27965_MOESM1_ESM.pdf]

Description of Supplementary Files

File Name: Supplementary Information for **"Intensified continental chemical weathering and carbon cycle perturbations linked to volcanism during the Triassic–Jurassic transition"** by Shen et al.

Description: Supplementary Notes (1-4), Supplementary Figures (1-6), Supplementary Table, and Supplementary References.

Supplementary Notes

Supplementary Note 1 | Correlations of the study sections

We correlated the two study sections based on the following considerations. First, detailed palynological zonations are available for both HJG[1, 2, 3, 4, 5, 6, 7] and QLX[8]. The end-Triassic extinction horizon and T–J boundary can be recognized based on abrupt turnovers of plants in each section (Supplementary Fig. 1). Furthermore, the palynological assemblage at the base of the Zhenzhuchong Formation at QLX can be correlated with that at the base of the Badaowan Formation at HJG—they share many miospore genera including *Dictyophyllidites*, *Concavisporites*, *Asseretospora*, *Cyathidites*, *Chasmatosporites*, and *Quadraeculina*[6, 8] (Supplementary Fig. 1). Furthermore, the palynostratigraphic framework of the QLX section was established mainly based on changes in the relative abundances of some important spore-pollen genera (e.g. *Dictyophyllidites*, *Concavisporites*, *Cyathidites*, *Asseretospora*, *Quadraeculina*, *Chasmatosporites*) and the occurrences of key species (e.g. *Lunzisporites lunzensis*, *Conbaculatisporites pauculus*, *Neoraistrickia taylorii*, *Lycopodiacidites rudis*, *Annulispora* spp., *Kyrtomisporis laevigatus*, *Classopollis* spp.). Significantly, both palynological assemblages record fern spore spikes composed of a few genera that have been documented from the Triassic–Jurassic transition interval globally[8]. Second, organic carbon isotope profiles for each section exhibit similar features, i.e., three negative excursions—the PCIE, ICIE, and MCIE (Fig. 2, Supplementary Fig. 1), that also permit correlations to other continental and marine T–J boundary sections[9]. Finally, studies of Milankovitch cyclicity have yielded high-resolution temporal frameworks for both HJG[10] and QLX[11] (Fig. 2, Supplementary Fig. 1).

Supplementary Note 2 | Host phases of Hg in the sediment

Robust use of Hg as a volcanic proxy requires an understanding of its sedimentary host phases. Most studies have assumed organic matter to be the dominant host of Hg, relying on mercury to total organic carbon ratios (Hg/TOC) to assess Hg enrichments in ancient sediments[12, 13, 14]. However, Hg also can be associated with the sulfide[14, 15, 16] or clay fractions of the sediment[14, 17, 18], complicating TOC normalization of Hg concentration data. The exact environmental controls on

Hg-hosting by various sedimentary phases are not well understood. Hg-S complexes have high stability constants and can dominate Hg speciation under reducing conditions where considerable reduced S is present in porewaters[15, 16]. Clay minerals are also capable of adsorbing Hg, leading to significant Hg enrichments in some shales[14, 17, 18].

The Hg concentration profile of the HJG section exhibits limited variation, mostly from 3 ppb to 101 ppb (Supplementary Fig. 2a). It generally exhibits low TOC (<0.5 wt.%) for mudstones but high TOC (>10 wt.%) for some coal samples (Supplementary Fig. 2b). It has low total sulfur content (<0.3 wt.%, Supplementary Fig. 2c) and relatively stable Al values (mostly 6-12 wt.%, Supplementary Fig. 2d). Hg concentrations do not exhibit significant covariation with any of these proxies (TOC, TS and Al; Supplementary Fig. 4), rendering unclear whether there is a dominant host phase of Hg in this section. Despite this uncertainty, we chose to utilize Hg/TOC normalizations rather than Hg/TS or Hg/Al normalizations, which has the advantage of maintaining equivalency of data display with the QLX section (in which Hg is unambiguously hosted mainly by the organic fraction of the sediment) as well as many other works during earth history (see reviews by Grasby et al.[13]). At HJG, the variability in Hg/TOC peaks near the ICIE may be partly due to low TOC values (Fig. 2c). It is possible that, given the low sampling density of this section (116 samples in ~650 m strata), we inadvertently failed to sample the T–J boundary beds with the highest TOC content. A higher-resolution study of HJG (61 samples in ~90 m strata) yielded higher Hg/TOC values around the T–J boundary[19], although the pattern of secular variation is much the same as in the present study. Given the similarity of Hg/TOC records from two independent groups, it appears that the Hg enrichment interval around the T–J boundary has been reliably identified in the present study as well.

At QLX, Hg concentrations vary strongly throughout the section, ranging from <10 ppb to >1000 ppb (Supplementary Fig. 3a). TOC also exhibits large variations, with lower values in the Upper Triassic but higher values near the T–J transition and in the Lower Jurassic (Supplementary Fig. 3b). Al contents are less variable, ranging from 3 wt.% to 13 wt.% (Supplementary Fig. 3c). Hg concentrations show strong positive covariation with TOC ($r = +0.76$, $n = 106$, $p < 0.01$) and modest negative covariation with Al ($r = –0.46$, $n = 106$, $p < 0.01$), suggesting that organic matter is the dominant host of Hg (Supplementary Fig. 4). For this reason, Hg/TOC is a suitable normalization for Hg at QLX. The TOC values for samples yielding higher Hg/TOC (e.g., >100 ppb/wt.%) range

from < 0.2 wt.% to 2.5 wt.%. The TOC values (0.19 wt.%, 0.15 wt.%, 0.16 wt.%, 0.18 wt.%, 0.18 wt.%, see Supplementary Dataset) are close to the threshold value (i.e., 0.2 wt.%) for five of the high Hg/TOC samples within the mercury enrichment interval (Figs. 2i, Supplementary Fig. 3), but high Hg/TOC values are also exhibited in samples with high TOC content. Thus, the pattern of the Hg/TOC profile at QLX would not be changed significantly by exclusion of these five samples.

Supplementary Note 3 | Chemical index of alteration

The A-CN-K diagram is widely used as a supplement to CIA for evaluation of weathering intensity[20]. First, it provides a means to evaluate the compositional consistency of source materials. Both experimental studies and analyses of geological samples show that the weathering of upper continental crust, which is mainly composed of plagioclase- and K-feldspar-rich rocks, features a leaching sequence in which depletion of Na_2O and CaO dominates the early stages of weathering, followed by accelerated loss of K_2O in a more advanced weathering stage[21]. An ideal weathering trend for a succession of compositionally homogenous first-cycle source materials would therefore consist of a subparallel line relative to the A-CN axis representing early-stage weathering which veers toward the A apex as weathering enters the more advanced stage. The intersection of the weathering trend with the CN-K join represents the relative ratios of plagioclase and K-feldspar in the source materials. Any discernible change of source material usually results in a deviation from this ideal weathering trend.

Second, the A-CN-K diagram demonstrates the extent to which samples have experienced potassium (K^+) metasomatism, a common post-burial alteration process that usually takes the form of illitization of clay minerals and/or authigenic formation of K-feldspars, and that causes deviations from the ideal weathering trend toward the K apex[20]. Once a stable and consistent sedimentary source supply is confirmed, corrections for the addition of potassium can be made by extrapolating each sample back to the ideal weathering trend on the A-CN-K diagram[20]. In the present study, samples from each section follow the trend of an ideal weathering sequence, which strongly supports a relatively consistent sediment source for each section (Fig. 4). Thus, A-CN-K diagrams provide no evidence for K metasomatism, and therefore no K correction was applied in this study.

Prior to calculation of CIA, study units should be evaluated for their suitability for

paleoweathering analysis through examination of source stability, grain-size uniformity, and dilution by autogenic components, as determined from independent proxies[22]. A proxy for source stability is the elemental ratio Al/Ti, which is governed primarily by its parent materials and remains relatively invariant under most weathering regimes given a stable source[21]. In the present study sections, no significant stratigraphic variations in Al/Ti were observed (Supplementary Figs. 2, 3 and 5), providing support for an invariant source of weathering materials to each section, as already inferred from A-CN-K relationships (Fig. 4). Although small differences exist between mean Al/Ti values and a noticeable horizontal shift of the initial weathering trends in both sections speak for differences in the source components (Supplementary Fig. 5), this has no impact on weathering interpretations of the two study sections. Grain-size influences on CIA can be examined using Al/Si ratios[23]. In the present study sections, Al/Si exhibits limited stratigraphic variations, ruling out a major influence of grain size on CIA (Supplementary Figs. 2, 3 and 5). Regarding autogenic mineral dilution, a WIP-CIA crossplot shows a near-linear negative correlation with little deviation for both study sections (Supplementary Fig. 6), consistent with insignificant influence by autogenic components[24]. As a precaution, we removed the CIA data for samples with high TOC (e.g., >18 wt.%) in the HJG section (see Supplementary Dataset). All factors considered, CIA can serve as a robust paleoweathering proxy in both study sections.

Supplementary Note 4 | LOSCAR run and sensitivity tests

Carbon cycle simulations for carbon emissions during the T–J transition were performed using the Long-term Ocean-Atmosphere-Sediment CArbon cycle Reservoir (LOSCAR) model[25]. This model is designed to calculate the partitioning of carbon between ocean, atmosphere, and sediments on various time scales and is suitable for simulating atmospheric $p\mathrm{CO_2}$ and surface-ocean carbon isotopes during and after carbon injection events over thousands to millions of years.

As the LOSCAR model was not specifically designed to simulate end–Triassic carbon cycle perturbations (for example, the end–Triassic world featured one dominant ocean—the Panthalassic Ocean—whereas the configuration of LOSCAR has either three or four oceans), we needed to modify specific parameters in the original LOSCAR model to reflect the critical end–Triassic boundary conditions[26]. For this reason, we used PETM boundary conditions instead of modern conditions to model the T–J boundary carbon cycle perturbations. Based on $p\mathrm{CO_2}$ estimates for the

end-Triassic[27, 28], we set the initial steady-state atmospheric pCO_2 at 2000 ppm. We increased the temperature of the low-latitude surface ocean to 32 °C[28, 29]. We updated the concentrations of dissolved Ca and Mg to 17 mM and 32 mM, respectively[30]. Also, as shown by previous studies using LOSCAR, variation in modeled $\delta^{13}C$ values for the surface layer of different oceans is small, indicating that the separate ocean boxes can reasonably be represented by a single dominant sea[26]. To make our simulations more robust, we turned on the climate sensitivity (i.e., the temperature change for a doubling of atmospheric CO_2 was set to 3 °C; TSNS changed to 1 in Supplementary Table 1) and increased *nsi* (i.e., the silicate weathering exponent) from a default value of 0.2 to a revised value of 0.3, to reconcile the underestimation of silicate weathering fluxes[26]. The detailed modifications to LOSCAR are shown in Supplementary Table 1.

The total amount of CO_2 released by the CAMP is assumed to be 24,000 Gt carbon, with an average $\delta^{13}C$ of −18.8 ‰[26]. Its release rate is assumed to have followed a Gaussian pattern during the 600-kyr-long eruption interval starting from model time zero[31, 32]. The total runtime was set to 5 million years. In view of additional global weathering rate changes linked to eruption of highly weatherable basalts, we multiplied the silicate weathering rate in LOSCAR with an additional weatherability term *k_silw*[33], which was either set to 1 through the whole run time (which was unlikely the real case) or increased linearly from 1.0 to a maximum value through the 600-kyr eruption interval and then allowed to decrease linearly back to 1.0 within 2 Myr following the eruption termination. As sensitivity tests, we set the maximum *k_silw* as 1.1, 1.2 and 1.3 in different runs. As shown in Figure 4, the atmospheric CO_2 levels and silicate weathering fluxes responded variously to the different *k_silw* values. When *k_silw* was fixed at 1, it took ~3 Myr for the system to be restored to its initial state following the carbon cycle perturbation. In contrast, when the maximum *k_silw* was set at 1.3, atmospheric pCO_2 returned to its initial 2000 ppm within ~1 Myr and then decreased further as a result of the higher *k_silw* value. Although actual *k_silw* values during the end-Triassic event cannot be precisely constrained, by comparing the timeframes of the atmospheric pCO_2 and silicate weathering responses derived from the LOSCAR model with the carbon-cycle excursion and weathering anomalies in our geochemical records (over a ~1.6 Myr interval), we concluded that only a very small increase in the weatherability of continental silicates (i.e., *k_silw* ~1.1) is necessary to drive the observed geochemical trends.

Supplementary References

1. Huang, P. Sporopollen assemblages from the Haojiagou and Badaowan formations at the Haojiagou section of Urumqi, Xinjiang and their stratigraphic significance. *Acta Micropal. Sin.* **23**, 235-274 (2006).
2. Lu, Y. & Deng, S. Palaeoclimate around the Triassic–Jurassic Boundary in southern margin of Junggar Basin. *J. Palaeogeog*. **11**, 652-660 (2009). (In Chinese with English abstract)
3. Sha, J. et al. Stratigraphy of the Triassic–Jurassic boundary successions of the southern margin of the Junggar Basin, northwestern China. *Acta Geol. Sin.* **85**, 421-436 (2011).
4. Sha, J. et al. Triassic–Jurassic climate in continental high-latitude Asia was dominated by obliquity-paced variations (Junggar Basin, Ürümqi, China). *Proc. Natl. Acad. Sci. U.S.A.* **112***,* 3624-3629 (2015).
5. Lu, Y. & Deng, S. Triassic–Jurassic sporopollen assemblages on the sourthern margin of the Junggar basin, Xinjiang and the T–J boundary. *Acta Geol. Sin.* **79**, 15-28 (2005).
6. Deng, S. et al. The Jurassic System of northern Xinjiang, China. *University of Science and Technology of China Press*, 1-279 (2010).
7. Zhang, J., Lenz, O. K., Hornung, J., Wang, P., Ebert, M. & Hinderer, M. Palynology and the Eco-Plant model of peat-forming wetlands of the Upper Triassic Haojiagou Formation in the Junggar Basin, Xinjiang, NW China. *Paleogeogr. Paleoclimatol. Paleoecol.* **556**, 109888 (2020).
8. Li, L., Wang, Y. D., Kürschner, W. M., Ruhl, M. & Vajda, V. Palaeoclimatology, Palaeoecology. Palaeovegetation and palaeoclimate changes across the Triassic–Jurassic transition in the Sichuan Basin, China. *Paleogeogr. Paleoclimatol. Paleoecol.* **556**, 109891 (2020).
9. Ruhl, M. et al. On the onset of Central Atlantic Magmatic Province (CAMP) volcanism and environmental and carbon-cycle change at the Triassic–Jurassic transition (Neuquén Basin, Argentina). *Earth-Sci. Rev.* **208**, 103229 (2020).
10. Shen, J. et al. Marine productivity changes during the end-Permian crisis and Early Triassic recovery. *Earth-Sci. Rev.* **149**, 136-162 (2015).
11. Li, M. et al. Astronomical tuning and magnetostratigraphy of the Upper Triassic Xujiahe

Formation of South China and Newark Supergroup of North America: Implications for the Late Triassic time scale. *Earth Planet. Sci. Lett.* **475**, 207-223 (2017).

12. Ravichandran, M. Interactions between mercury and dissolved organic matter—a review. *Chemosphere* **55**, 319-331 (2004).
13. Grasby, S. E., Them II, T. R., Chen, Z., Yin, R. & Ardakani, O. H. Mercury as a proxy for volcanic emissions in the geologic record. *Earth-Sci. Rev.* **196**, 102880 (2019).
14. Shen, J. et al. Sedimentary host phases of mercury (Hg) and implications for use of Hg as a volcanic proxy. *Earth Planet. Sci. Lett.* **543**, 116333 (2020).
15. Bower, J., Savage, K. S., Weinman, B., Barnett, M. O., Hamilton, W. P. & Harper, W. F. Immobilization of mercury by pyrite (FeS_2). *Environ. Pollut.* **156**, 504-514 (2008).
16. Shen, J. et al. Mercury in marine Ordovician/Silurian boundary sections of South China is sulfide-hosted and non-volcanic in origin. *Earth Planet. Sci. Lett.* **511**, 130-140 (2019).
17. Farrah, H. & Pickering, W. F. The sorption of mercury species by clay minerals. *Water Air Soil Poll.* **9**, 23-31 (1978).
18. Shen, J. et al. Mercury evidence of intense volcanic effects on land during the Permian-Triassic transition. *Geology* **47**, 1117-1121 (2019).
19. Zhang, X. et al. Wildfire records across the Triassic–Jurassic Boundary in the Southern margin of the Junggar Basin. *Acta Sedimentol. Sin.* (2021). Doi: 10.14027/j.issn.1000-0550.2020.103. (In Chinese with English abstract)
20. Fedo, C. M., Nesbitt, W. H. & Young, G. M. Unraveling the effects of potassium metasomatism in sedimentary rocks and paleosols, with implications for paleoweathering conditions and provenance. *Geology* **23**, 921-924 (1995).
21. Panahi, A., Young, G. M. & Rainbird, R. H. Behavior of major and trace elements (including REE) during Paleoproterozoic pedogenesis and diagenetic alteration of an Archean granite near Ville Marie, Quebec, Canada. *Geochim. Cosmochim. Ac.* **64**, 2199-2220 (2000).
22. Grygar, T. M., et al. Lithological correction of chemical weathering proxies based on K, Rb, and Mg contents for isolation of orbital signals in clastic sedimentary archives. *Sediment. Geol.* **406**, 105717 (2020).
23. Grygar, T. M., Mach, K. & Martinez, M. Checklist for the use of potassium concentrations in siliciclastic sediments as paleoenvironmental archives. *Sediment. Geol.* **382**, 75-84 (2019).

24. Garzanti, E., Padoan, M., Setti, M., Najman, Y., Peruta, L. & Villa, I. M. Weathering geochemistry and Sr-Nd fingerprints of equatorial upper Nile and Congo muds. *Geochem. Geophys. Geosyst*. **14**, 292-316 (2013).

25. Zeebe, R. E. LOSCAR: Long-term ocean-atmosphere-sediment carbon cycle reservoir model v2.0.4. *Geosci. Model Dev.* **5**, 149-166 (2012).

26. Heimdal, T. H., Jones, M. T. & Svensen, H. H. Thermogenic carbon release from the Central Atlantic magmatic province caused major end–Triassic carbon cycle perturbations. *Proc. Natl. Acad. Sci. U.S.A* **117**, 11968-11974 (2020).

27. McElwain, J. C., Beerling, D. J. & Woodward, F. I. Fossil plants and global warming at the Triassic-Jurassic boundary. *Science* **285**, 1386-1390 (1999).

28. Schaller, M. F., Wright, J. D. & Kent, D. V. Atmospheric pCO_2 perturbations associated with the Central Atlantic magmatic province. *Science* **331**, 1404-1409 (2011).

29. Steinthorsdottir, M., Jeram, A. J. & McElwain, J. C. Extremely elevated CO_2 concentrations at the Triassic/Jurassic boundary. *Paleogeogr. Paleoclimatol. Paleoecol.* **308**, 418-432 (2011).

30. Horita, J., Zimmermann, H. & Holland, H. D. Chemical evolution of seawater during the Phanerozoic: Implications from the record of marine evaporites. *Geochim. Cosmochim. Ac.* **66**, 3733-3756 (2002).

31. Blackburn, T. J. et al. Zircon U-Pb geochronology links the end-Triassic extinction with the Central Atlantic Magmatic Province. *Science* **340**, 941-945 (2013).

32. Davies, J. H. F. L., Marzoli, A., Bertrand, H., Youbi, N., Ernesto, M. & Schaltegger, U. End-Triassic mass extinction started by intrusive CAMP activity. *Nat. Commun.* **8**, 15596 (2017).

33. Caves, J. K., Jost, A. B., Lau, K. V. & Maher, M. K. Cenozoic carbon cycle imbalances and a variable weathering feedback. *Earth Planet. Sci. Lett.* **450**, 152-163 (2016).

Supplementary Figures:

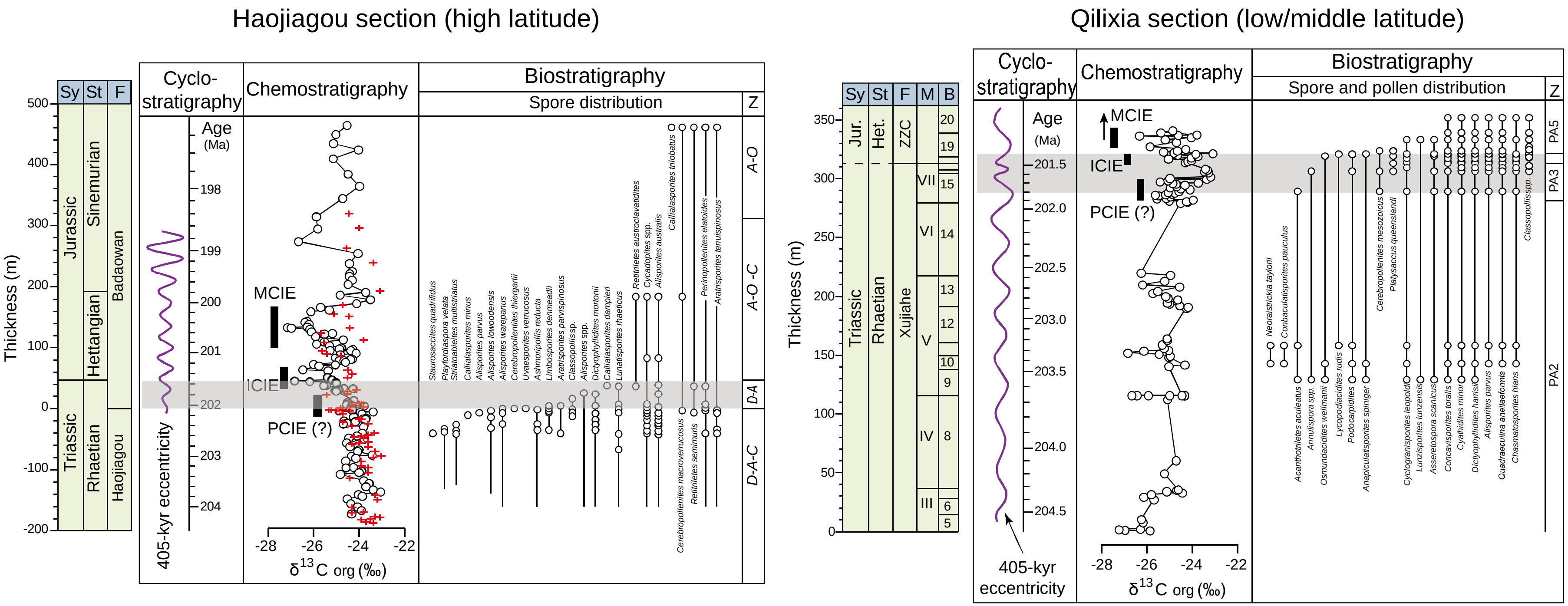

Supplementary Figure 1. Correlations between the Haojiagou section (high latitude) and the Qilixia section (low/middle latitude) based on cyclostratigraphic analysis (Haojiagou: Sha et al.[4]; Qilixia: Li et al.[11]), chemostratigraphy (open circles from this study; the red cross at HJG from Sha et al.[4]), and biostratigraphy (Haojiagou: Sha et al.[4]; Qilixia: Li et al.[8]). The gray rectangles represent the plants turnover interval. Other details as in Figure 2.

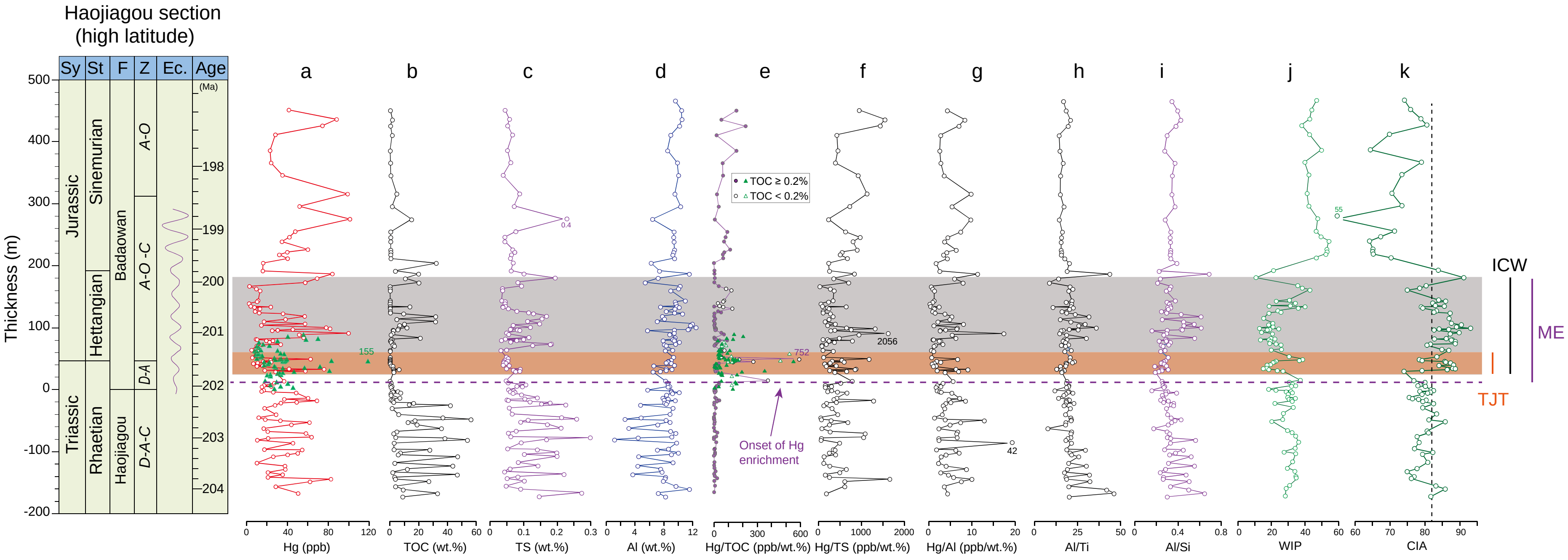

Supplementary Figure 2. Profiles of Haojiagou section: (a) mercury concentration (Hg, ppb); (b) total organic carbon concentration (TOC, wt.%); (c) total sulfur concentration (TS, wt.%); (d) aluminum concentration (Al, wt.%); (e) ratio of mercury to total organic carbon (Hg/TOC, ppb/wt.%); (f) ratio of mercury to total sulfur (Hg/TS, ppb/wt.%); (g) ratio of mercury to aluminum (Hg/Al, ppb/wt.%); (h) ratio of aluminum to titanium (Al/Ti); (i) ratio of aluminum to silicon (Al/Si); (j) Weathering Index of Parker (WIP); and (k) Chemical Index of Alteration (CIA). WIP = 100 × [$(2Na_2O/0.35) + (MgO/0.9) + (2K_2O/0.25) + (CaO^*/0.7)$], where CaO* represents the corrected CaO in silicate minerals following the method of Fedo et al.[20]. The green triangles in a and e represent the Hg and Hg/TOC for HJG, respectively, from Zhang et al. [47]. ICW = interval of intense chemical weathering; TJT = Triassic–Jurassic transition; ME = mercury-enriched interval. Other details as in Figure 2. Source data are provided as a Source Data file

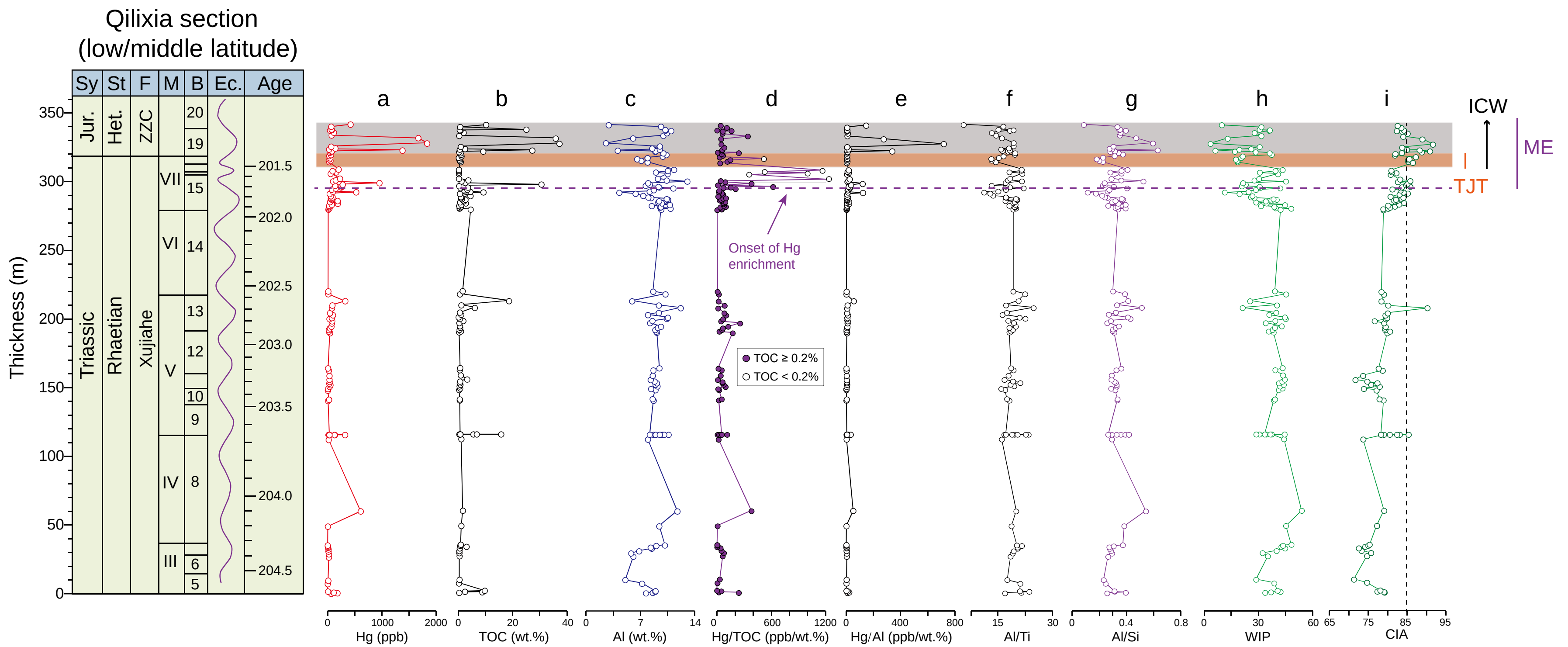

Supplementary Figure 3. Profiles of Qilixia section: (a) mercury concentration (Hg, ppb); (b) total organic carbon concentration (TOC, wt.%); (c) aluminum concentration (Al, wt.%); (d) ratio of mercury to total organic carbon (Hg/TOC, ppb/wt.%); (e) ratio of mercury to aluminum (Hg/Al, ppb/wt.%); (f) ratio of aluminum to titanium (Al/Ti); (g) ratio of aluminum to silicon (Al/Si); (h) Weathering Index of Parker (WIP); and (i) Chemical Index of Alteration (CIA). ICW = interval of intense chemical weathering; TJT = Triassic–Jurassic transition; ME = mercury-enriched interval. Other details as in Figures 2, S1, and S2. Source data are provided as a Source Data file

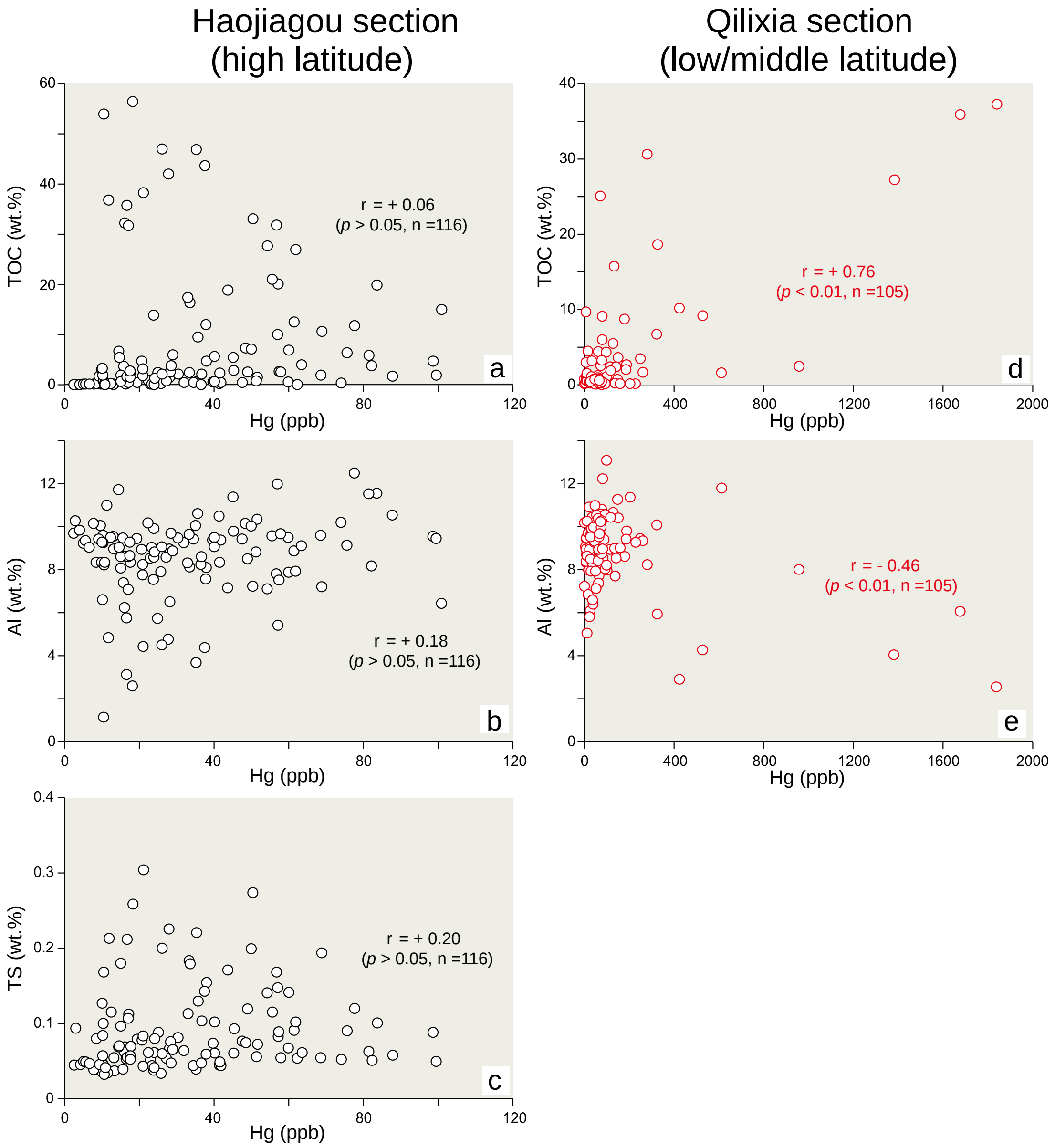

Supplementary Figure 4. Crossplots of TOC vs Hg, Al vs Hg, and TS vs Hg for the Haojiagou section (a, b, c; black symbols), and the Qilixia section (d, e; red symbols; note: no TS data). Source data are provided as a Source Data file

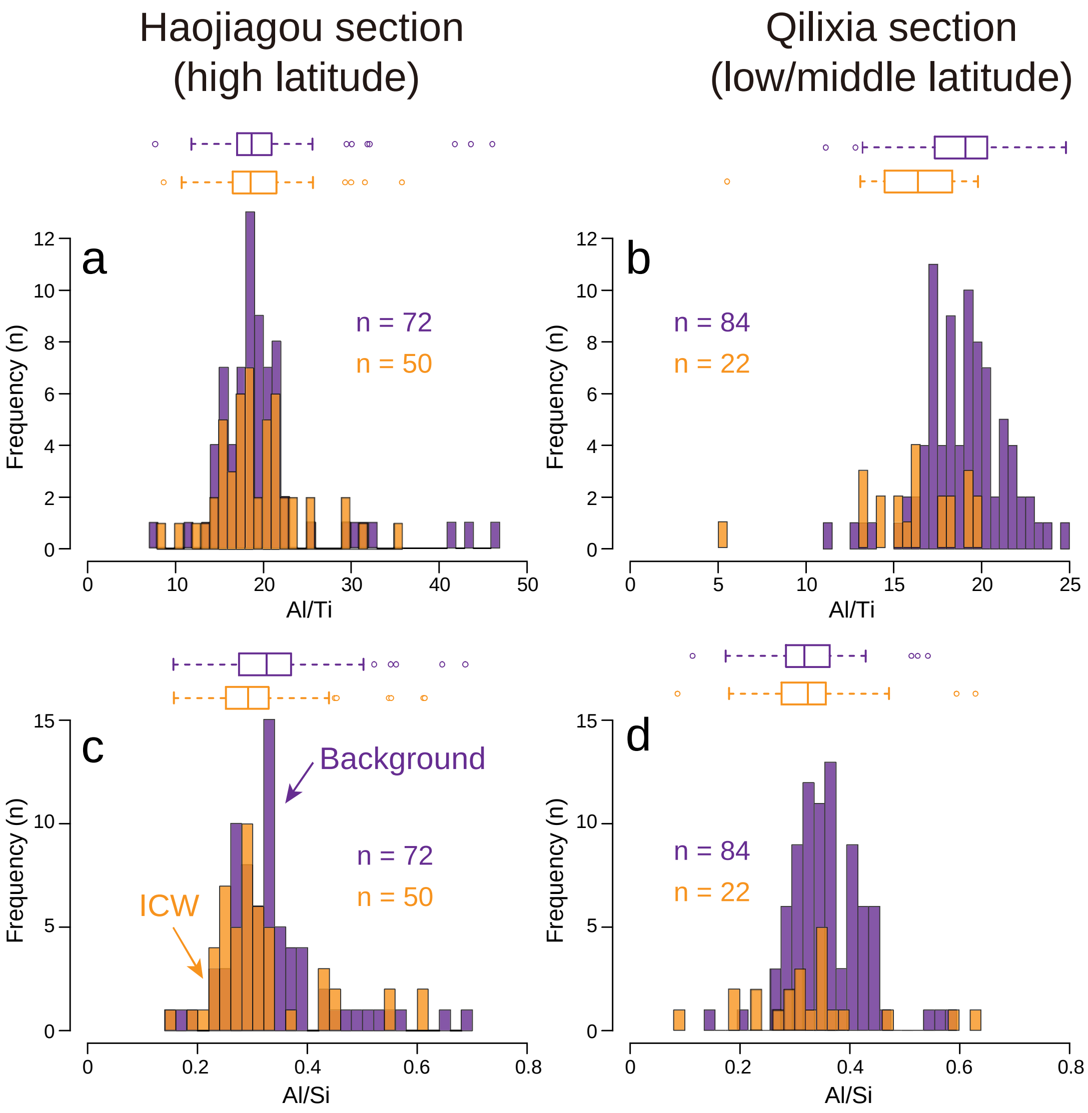

Supplementary Figure 5. Histogram and boxplot of ratios of aluminum to titanium (Al/Ti) and aluminum to silicon (Al/Si) for Haojiagou (a, c) and Qilixia (b, d). *n* represents the number of samples from intensely chemically weathered (ICW; orange) and background intervals (purple). Source Data are provided as a Source Data file

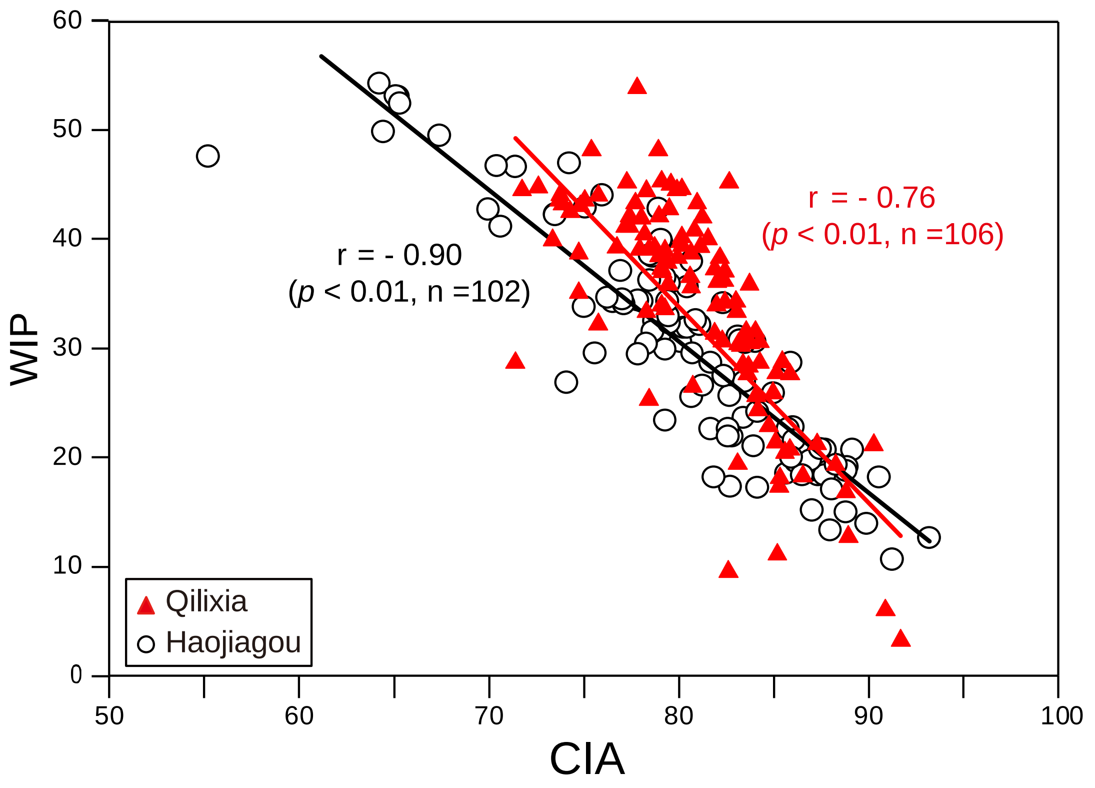

Supplementary Figure 6. Crossplot between Weathering Index of Parker (WIP) and Chemical Index of Alteration (CIA) for Haojiagou (open circles) and Qilixia (red triangles). Source data are provided as a Source Data file

Supplementary Table 1 Modified LOSCAR parameters

Parameter	Default	New value	Unit	Description
TSNS	0	1		Switch temperature sensitivity to pCO_2 (0=OFF, 1=ON)
TEMP (surface)	25	32	℃	Temperature all low latitude surface boxes
PCO2SI	1000	2000	ppm	Initial steady-state pCO_2
CALC	0.02	0.017	mol/kg	Seawater Ca^{2+}
MAGN	0.03	0.032	mol/kg	Seawater Mg^{2+}
nsi	0.2	0.3		Silicate weathering exponent
