# Supplementary material for: Intensified continental chemical weathering and carbon-cycle perturbations linked to volcanism during the Triassic–Jurassic transition
Source: Nat Commun. 2022 Jan 13;13:299. doi: 10.1038/s41467-022-27965-x (PMC8758789; doi:10.1038/s41467-022-27965-x)
Supplement: Supplementary file 3 — Description of Additional Supplementary Files [file 41467_2022_27965_MOESM3_ESM.pdf]

Description of Additional Supplementary Files

File Name: Supplementary Data 1
Description: Geochemical datasets for the two study sections.
